# Evidence That *ITPR2*-Mediated Intracellular Calcium Release in Oligodendrocytes Regulates the Development of Carbonic Anhydrase II + Type I/II Oligodendrocytes and the Sizes of Myelin Fibers

**DOI:** 10.3389/fncel.2021.751439

**Published:** 2021-09-22

**Authors:** Ruyi Mei, Linyu Huang, Mengyuan Wu, Chunxia Jiang, Aifen Yang, Huaping Tao, Kang Zheng, Junlin Yang, Wanhua Shen, Xianjun Chen, Xiaofeng Zhao, Mengsheng Qiu

**Affiliations:** ^1^College of Life Sciences, Zhejiang University, Hangzhou, China; ^2^Zhejiang Key Laboratory of Organ Development and Regeneration, College of Life and Environmental Sciences, Institute of Developmental and Regenerative Biology, Hangzhou Normal University, Hangzhou, China; ^3^Department of Physiology, Research Center of Neuroscience, Chongqing Medical University, Chongqing, China

**Keywords:** axon diameter, conduction velocity, oligodendrocyte, myelination, *ITPR2*

## Abstract

Myelination of neuronal axons in the central nervous system (CNS) by oligodendrocytes (OLs) enables rapid saltatory conductance and axonal integrity, which are crucial for normal brain functioning. Previous studies suggested that different subtypes of oligodendrocytes in the CNS form different types of myelin determined by the diameter of axons in the unit. However, the molecular mechanisms underlying the developmental association of different types of oligodendrocytes with different fiber sizes remain elusive. In the present study, we present the evidence that the intracellular Ca^2+^ release channel associated receptor (*Itpr2)* contributes to this developmental process. During early development, *Itpr2* is selectively up-regulated in oligodendrocytes coinciding with the initiation of myelination. Functional analyses in both conventional and conditional *Itpr2* mutant mice revealed that *Itpr2* deficiency causes a developmental delay of OL differentiation, resulting in an increased percentage of CAII^+^ type I/II OLs which prefer to myelinate small-diameter axons in the CNS. The increased percentage of small caliber myelinated axons leads to an abnormal compound action potentials (CAP) in the optic nerves. Together, these findings revealed a previously unrecognized role for *Itpr2*-mediated calcium signaling in regulating the development of different types of oligodendrocytes.

## Introduction

In the central nervous system (CNS), myelin is elaborated by oligodendrocytes (OLs) and plays a crucial role in axonal conductance and integrity (Fields, 2008; Martins-de-Souza, 2010; Nave, 2010; Miron et al., 2011; Edgar and Sibille, 2012). Abnormal myelin development has been implicated in several neuropsychiatric diseases including schizophrenia and major depression (Fields, 2008; Martins-de-Souza, 2010; Edgar and Sibille, 2012), and defective motor skill learning (McKenzie et al., 2014). Previous studies demonstrated that not all axons in the CNS are myelinated, and in general, only larger axons of certain diameters (>0.2 μm) are ensheathed by oligodendrocyte processes. In 1928, del Río Hortega classified the oligodendrocytes into four types (type I–type IV) according to their morphological features (HP, 1928). Among these four types, type I/II oligodendrocytes predominantly myelinate small diameter axons (0.2–0.4 μm), whereas type III/IV oligodendrocytes that myelinate larger caliber axons (Butt et al., 1995). More recent studies showed that isoenzyme carbonic anhydrase II (CAII) is a specific marker for type I/II oligodendrocytes, while type III/IV oligodendrocytes are CAII-negative (Butt et al., 1998; Butt and Berry, 2000). Intriguingly, oligodendrocytes appear to be very plastic and can change their phenotype, as oligodendrocytes that normally myelinate small diameter axons are able to myelinate large diameter axons when transplanted into demyelinated tracts (Fanarraga et al., 1998), and expression of CAII increases when the volume of supported myelin decreases (O’Leary and Blakemore, 1997; Berry et al., 1998). Although recent single-cell sequencing analyses have provided additional molecular evidence for the existence of different types of oligodendroglia (Zeisel et al., 2015; Marques et al., 2016), the molecular mechanisms underlying the development of oligodendrocyte subpopulations and their association with different fiber sizes have remained elusive.

Early studies have suggested that intracellular calcium signaling plays an important role in the survival and differentiation of oligodendrocyte progenitor cells (OPCs), and the maintenance of the myelin sheath as well (Soliven, 2001; Paez et al., 2012). The calcium homeostasis imbalance can result in demyelinating disease (Tsutsui and Stys, 2013). Although oligodendrocytes can release Ca^2+^ from internal stores through both inositol 1,4,5-trisphosphate receptors (ITPRs) and ryanodine receptors (RyRs), only ITPRs could evoke the Ca^2+^ waves in newly differentiated OLs and initiate the myelin formation process (Haak et al., 2001). ITPRs are intracellular Ca^2+^ release channels that are mainly localized in the endoplasmic reticulum (ER). There are three isoforms of ITPRs (ITPR1-3) that are differentially expressed in the CNS tissues, with ITPR2 being solely transcribed in glial cells (Sharp et al., 1999). However, the expression and functional involvement of *Itpr2* in oligodendrocyte development and myelinogenesis has not been defined.

In this study, we report that *Itpr2* is selectively upregulated in oligodendrocytes during differentiation and myelin formation stages. Functional studies with both conventional and conditional mutants revealed that *Itpr2* deficiency causes a developmental delay of oligodendrocyte differentiation in the CNS, resulting in an increased percentage of CAII^+^ type I/II OLs and small-diameter myelinated axons with abnormal CAP.

## Materials and Methods

### Animals

All animal experiments were performed in accordance with the institutional guidelines drafted by the Laboratory Animal Center, Hangzhou Normal University, and were approved by the Animal Ethics Committee of Hangzhou Normal University, China. The *Itpr2-KO, Itpr2^*flox*^, Myrf^*flox*^, Nkx2.2^*flox*^, Olig1*-Cre, *Cnp-*Cre, and *Sox10-GFP* mouse lines were described previously (Lu et al., 2002; Lappe-Siefke et al., 2003; Li et al., 2005; Emery et al., 2009; Tripathi et al., 2011; Mastracci et al., 2013). For the removal of *Itpr2* in oligodendrocyte lineage, *Itpr2^*flox*^* mice were interbred with *Cnp*-Cre transgenic mice to confirm that the myelination phenotypes observed in *Itpr2* conventional knockouts are attributable to oligodendrocyte-specific defects. Animals of either sex were used for analyses.

### Electron Microscopy

Wild type and mutant mice perfused with a phosphate buffer solution containing 2.5% glutaraldehyde and 4% paraformaldehyde (PFA, pH 7.2). The optic nerve and corpus callosum tissues were isolated and post-fixed in 1% osmium tetroxide for 1 h. Tissues were then washed in 0.1 M cacodylate buffer, dehydrated in graded ethanol and embedded in epoxy resins. Ultrathin sections (0.5 μm) were stained with toluidine blue and observed under a transmission electronic microscope.

### Electrophysiology

All experiments were performed at room temperature (22–25°C). During preparation, artificial cerebrospinal fluid (aCSF) containing (in mM): NaCl 126, KCl 3.0, CaCl_2_ 2.0, MgCl_2_ 2.0, NaH_2_PO_4_ 1.2, NaHCO_3_ 26, and glucose 10, was continuously equilibrated with a humidified gas mixture of 95% O_2_/5% CO_2_. The optic nerves were dissected out at the optic chiasm behind the orbit. The nerve tissues were equilibrated in the beaker with aCSF for 30 min before each experiment. Recording micropipettes were pulled from borosilicate glass capillaries and the glass nozzles were polished until they could adhere to the optic nerves tightly. One micropipette was attached to the rostral end of the nerve for stimulation, the end of which was held by a custom-made stimulating suction electrode, which was made of a polished glass wrapped with silver wires and controlled by an isolator. The second micropipette was attached to the caudal end of the nerve for recording, and all recordings were orthodromic. The maximum compound action potentials (CAP) were evoked with electrical pulses at 0.05 ms in duration elicited at 0.2 Hz. While this process was completed, the stimulus pulse intensity was reduced to evoke 70% maximum reaction and recorded for 20 min. Signals were filtered at 2 kHz with a MultiClamp 700B amplifier (Molecular Devices, Palo Alto, CA). Data were sampled at 10 kHz and analyzed using ClampFit 10 (Molecular Devices). The curve fitting routine for describing the CAP in terms of Gaussian functions has previously been described (Allen et al., 2006), and data were fit using Microsoft Excel. The stimulus artifact was included in the fitting procedure as it impinges upon the 1st CAP peak. The best fit of a CAP by multiple Gaussian functions provides parameters that can be used to reconstruct the CAP.

### *In situ* RNA Hybridization

Tissues were fixed with 4% PFA in PBS (pH 7.4) at 4°C overnight. Tissues were then cryo-protected in 30% sucrose, embedded in optimal cutting temperature compound (OCT) medium, and sectioned on a cryostat at 16–18 μm. The procedures for *in situ* hybridization (ISH) have been described previously (Zhu et al., 2013). The digoxin-labeled RNA probes used for ISH corresponded to nucleotides 1210–2178 of mouse *Plp1* mRNA (NM_011123.4), nucleotides 7028–7970 of mouse *Itpr1* mRNA (NM_010585.5), nucleotides 134–698 of mouse *Itpr2* mRNA (NM_019923.4), and nucleotides 6818–7761 of mouse *Itpr3* mRNA (NM_080553.3).

### Immunofluorescence Staining

Animals were fixed by transcardial perfusion with cold 4% PFA after the animals were deeply anesthetized. Brains, spinal cords and optic nerves were isolated and post fixed overnight, cryoprotected in 30% sucrose, embedded in OCT compound and stored at −80°C for cryo-sectioning. After incubation in blocking buffer (10% goat serum and 0.2% Triton X-100 in PBS), tissue sections (16 μm thickness) were first incubated with primary antibodies at 4°C overnight and then with second antibodies at room temperature for 2 h, followed with 1 mg/mL DAPI for 5–10 min. Slides were mounted with mowiol mounting medium. The primary antibodies were used as follows: anti-OLIG2 (1:1,000, Millipore, Cat# AB9610, RRID: AB_570666), anti-CC1 (1:500, Abcam, Cat# ab16794, RRID: AB_443473), anti-ITPR2 (1:10, Millipore, Cat# AB3000, RRID: AB_91282), anti-NeuN (1:500, R and D Systems, Cat# MAB377, RRID: AB_2298767), anti-CAII (1:50, ABclonal, Cat# A1440, RRID: AB_2761269), anti-SOX10 (1:400, Oasis Biofarm), anti-ALDH1L1 (1:200, Oasis Biofarm). The secondary antibodies used were Alexa Fluor 488/594-conjugated antibodies (Invitrogen, Carlsbad, CA, United States).

### Western Blotting

Brainstem tissues were lysed in lysis buffer (Sigma, R0278) with protease inhibitor cocktail (Sigma, P8340). Proteins from control and mutant mice (30 μg each) were loaded for SDS-PAGE electrophoresis and subsequently detected with anti-ERK1/2 (1:5,000, Abcam, Cat# ab184699, RRID: AB_2802136), anti-Phospho-ERK1/2 (1:5,000, Abcam, Cat# ab76299, RRID: AB_1523577), anti-CNPase (1:2,000, Abcam, Cat# ab6319, RRID: AB_2082593), anti-β-Actin (1:10,000, ABclonal, Cat# AC026, RRID: AB_2768234) antibodies according to the protocol (Ray et al., 2000).

### Organotypic Slice Cultures

OL lineage-specific reporter mice *Sox10-GFP* at postnatal day 10 were sacrificed by cervical dislocation and then decapitated. Coronal slices (230 μm thick) from the mouse cerebral cortex were first sectioned in aCSF (pH 7.4), transferred onto 30 mm diameter semiporous membrane inserts (Millicell-CM PICM03050) and then cultured in six-well tissue culture dishes containing 3 mL of culture medium per well. The brain slice culture medium consisted of 50% Eagle’s minimal essential medium, 25% heat-inactivated horse serum, 25% Hank’s balanced salt solution, 1% L-glutamine, and 1% penicillin/streptomycin. Slices were maintained at 37°C in an incubator in atmosphere of humidified air and 5% CO_2_. After 1 day in culture, intracellular calcium chelator BAPTA-AM (20 μm) (Solarbio, S1102) was added to the culture medium to assess its impact on oligodendrocyte differentiation. For localization studies, the slices were fixed with 4% PFA for 24 h at 4°C after 5 days in culture and then detected the expression of CAII.

### Statistical Analysis

Data statistical analyses were performed using the GraphPad Prism software (version 8.0.2). For the quantitative analysis of the distribution of axonal size in the corpus callosum and optic nerve tissues, data were measured with a two-way analysis of variance (ANOVA) followed by a *post hoc* holm-sidak test. For the other data, one-way ANOVA followed by Sidak’s test was used for comparison among three groups, and unpaired *t*-test was used for comparison among two groups. All the error bars represent mean ± standard error of the mean (SEM) unless specified otherwise. And *p*-value < 0.05 was considered as statistically significant. For each analysis, the results from independent animals were treated as biological replicates (*n* ≥ 3).

## Results

### *Itpr2* Is Selectively Up-Regulated in Newly Differential Oligodendrocytes

**FIGURE 1:**
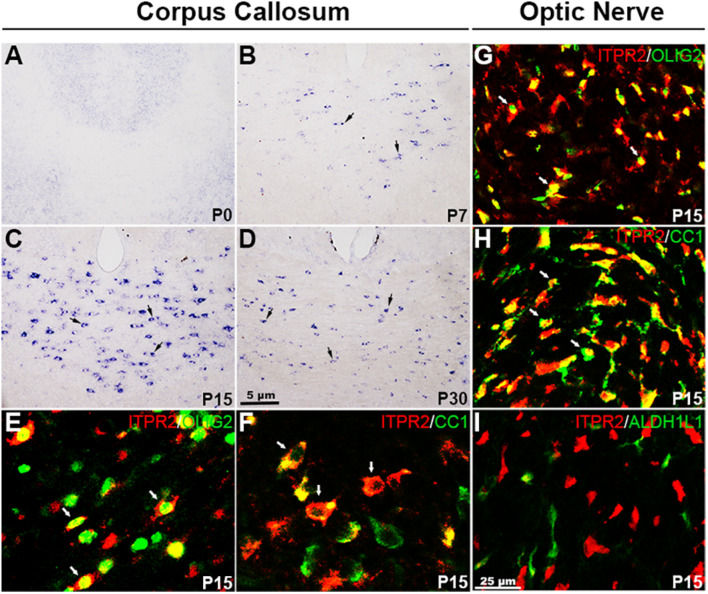
*Itpr2* is selectively up-regulated in differentiating OLs in the CNS. **(A–D)** Expression of *Itpr2* in the forebrain tissues as detected by RNA *in situ* hybridization (ISH). Black arrows highlight *Itpr2*^+^ cells. The number of *Itpr2* positive cells in the corpus callosum increased progressively from P7 to P15 and then decreased gradually thereafter. Scale bar represents 5 μm. **(E,F)** Double immunofluorescence of anti-ITPR2 combined with anti-OLIG2 or anti-CC1 in P15 corpus callosum. Double positive cells are represented by white arrows. CC, corpus callosum; CTX, Cortex. **(G–I)** Representative images of ITPR2^+^ cells that co-express OLIG2, CC1 but not ALDH1L1 in P15 optic nerve. Scale bar represents 25 μm.

A recent study suggested that *Itpr2* is strongly expressed in postnatal oligodendrocytes (Zeisel et al., 2015; Marques et al., 2016). To determine the specificity and developmental stages of *Itpr2* expression during oligodendrocyte development, we first performed RNA *in situ* hybridization (ISH) in the CNS tissues from different developmental stages. In the brain region, *Itpr2* expression started to emerge in the corpus callosum (CC) at around postnatal day (P7) stage, increased progressively thereafter (Figures 1A,B). By P15, its expression was detected throughout the entire CC tissue (Figure 1C). However, at P30, its expression was significantly down-regulated (Figure 1D). Similarly, in the spinal cord region, *Itpr2* was detected in the white matter glial cells starting at about embryonic day 18.5 (E18.5), and the number of *Itpr2*^+^ cells gradually increased with time and reached the maximum at P3-P7 stages (Supplementary Figures 1A–D). At later postnatal stages, *Itpr2* expression was gradually diminished in the white matter of spinal cord (Supplementary Figures 1E,F). The spatiotemporal pattern of *Itpr2* expression suggests its selective up-regulation in differentiating OLs (Figures 1A–D and Supplementary Figures 1A–F).

To further confirm that *Itpr2* is indeed expressed in differentiating OLs, we next examined the expression of *Itpr2* in the spinal cords of *Cnp^*cre/*+^; Nkx2.2^*fl/fl*^* and *Olig^cre/+^; Myrf^*fl/fl*^* mice. In the *Olig1*-Cre mouse line, the Cre activity is expressed in OPCs and mature OLs (Lu et al., 2002), whereas in the *Cnp-*Cre mouse line, the Cre is primarily expressed in early differentiating OLs (Yu et al., 1994; Baumann and Pham-Dinh, 2001). The *Cnp^*cre/*+^; Nkx2.2^*fl/fl*^* conditional mutant mice delayed OPC differentiation in the spinal cord (Q. Zhu et al., 2014), while *Olig^cre/+^; Myrf^*fl/fl*^* mice arrested oligodendrocyte differentiation and myelin gene expression (Emery et al., 2009; McKenzie et al., 2014; Xiao et al., 2016). As expected, the number of *Itpr2*^+^ cells was dramatically reduced in both *Nkx2.2* and *Myrf* conditional knock-out mice compared to the control groups (Supplementary Figure 2).

The selective expression of ITPR2 in differentiating OLs was further validated by double immunostaining with two well-established oligodendrocyte markers, CC1 and OLIG2, in P15 brain. Indeed, the majority of ITPR2-positive cells were co-stained with the newly formed OL marker CC1 (Cai et al., 2010; Young et al., 2013) or the general oligodendrocyte lineage marker OLIG2 in the corpus callosum (Figures 1E,F) and the optic nerves (Figures 1G,H). In addition, ITPR2^+^ cells did not co-express the astrocyte lineage marker ALDH1L1 in the optic nerves at early postnatal stage (Figure 1I). Similarly, ITPR2^+^ cells in P4-P7 spinal tissues (white matter) also co-expressed OLIG2 and CC1 but not with neuronal marker NeuN (Supplementary Figures 1G–J). Together, these data manifest that *Itpr2* is highly and selectively expressed in differentiating OLs in early postnatal CNS tissues, suggestive of its important role in OL maturation and myelination.

### Delayed Oligodendrocyte Differentiation in *Itpr2* Mutant Brain

**FIGURE 2:**
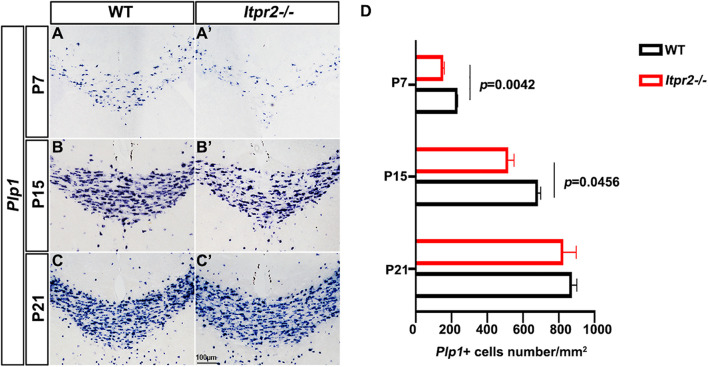
Ablation of *Itpr2* gene delays the differentiation of oligodendrocytes. **(A–C′)** *Plp1* mRNA expression in coronal forebrain sections of wild-type **(A–C)** and *Itpr2*-*KO* **(A′–C′)** mice at indicated stages. **(D)** Quantification of *Plp1*^+^ cells per section (*n* = 3) in the corpus callosum of *Itpr2KO* mice. Error bars indicate Means ± SEM. Scale bar represents 100 μm.

To assess the *in vivo* role of *Itpr2* in regulating OLs differentiation and myelin development, we next examined the expression of mature OL marker *Plp1* in postnatal brain tissues by ISH. It was found that the number of *Plp1*^+^ myelinating OLs in *Itpr2^–/–^* corpus callosum was significantly lower than that of controls between P7 and P15 stages (Figures 2A–B′,D) when OLs undergo active differentiation and myelination in this region (Franco-Pons et al., 2006; Korrell et al., 2019). Intriguingly, the number of *Plp1*^+^ OLs was not altered in P21 control and mutant tissues (Figures 2C,C′,D). These results indicate a transient developmental delay of OL differentiation when *Itpr2* gene is inactivated.

### Increased Percentage of Small Diameter Myelinated Axons in *Itpr2* Mutants

**FIGURE 3:**
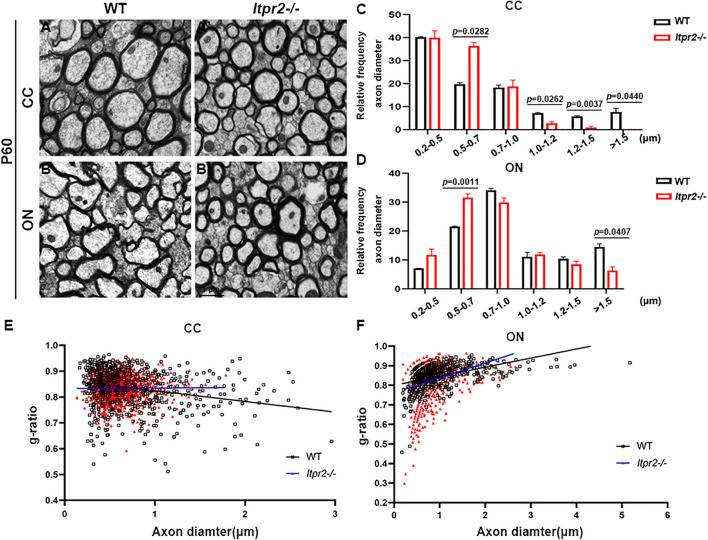
Knockout *Itpr2* increases the percentage of small-diameter myelinated axons in corpus callosum and optic nerve. **(A–B′)** Electron microscopy images of the corpus callosum (CC) **(A,A′)** and optic nerve (ON) **(B,B′)** from P60 wild-type and *Itpr2^–/–^* mice. Scale bar represents 1 μm. **(C)** Quantification of percentage of myelinated axons in the corpus callosum by size. An increased number of myelinated small-diameter axons (<0.7 μm) and a decreased number of myelinated large-diameter axons (>1.5 μm) were observed in *Itpr2* mutants. Two-way ANOVA *P*-values summary: interaction: *F* = 4.720, *p* = 0.0036; axon diameter: *F* = 36.29, *p* = 0.0010; genotype: *F* = 0.7167, *p* = 0.4359. **(D)** The distribution of axonal size in optic nerve is similar to that in the corpus callosum. Two-way ANOVA *P*-values summary: interaction: *F* = 11.61, *p* < 0.0001; axon diameter: *F* = 111.1, *p* < 0.0001; genotype: *F* = 0.5946, *p* = 0.4837. **(E)** The relationship between diameters and g-ratios of axons from the corpus callosum of wild-type and *Itpr2KO* mice at P60. Averaged g-ratios were 0.836 ± 0.007 (wild-type, 654 axons from 3 animals, black squares) and 0.833 ± 0.008 (*Itpr2*^–/^*^–^*, 1,447 axons from 4 animals, red triangles), *p* = 0.72. **(F)** The relationship between diameters and g-ratios of axons from the optic nerve of wild-type and *Itpr2KO* mice at P60. Averaged g-ratios were 0.858 ± 0.001 (wild-type, 635 axons from 3 animals, black squares) and 0.825 ± 0.070 (*Itpr2*^–/^*^–^*, 696 axons from 3 animals, red triangles), *p* = 0.55. Error bars indicate Means ± SEM.

To examine the effects of ITPR2 ablation on axonal myelination, we performed transmission electron microscopy (TEM) analysis on cross-sections of the corpus callosum (CC) and the optic nerve (ON) of postnatal day 60 (P60) wild-type and *Itpr2^–/–^* mice (Figures 3A–B′). Strikingly, ultrastructural analyses showed that there were significantly more myelinated axons in CC of *Itpr2^–/–^* mice compared with the controls. And optic nerve tissues showed a similar number of myelinated axons between wild-type and *Itpr2^–/–^* mice (Supplementary Figure 5). To evaluate whether axons of a certain caliber were more severely affected in the absence of ITPR2, we quantified the relative frequency of myelinated axons with respect to their corresponding diameters. Quantitative analyses showed that a larger proportion of myelinated axons had small diameters (0.2–0.7 μm) in the CC region of mutants, and the percentage of myelinated axons with large diameters was markedly reduced (larger than 1.5 μm) (Figure 3C; Two-way ANOVA *P*-values summary: interaction: *F* = 4.720, *p* = 0.0036; axon diameter: *F* = 36.29, *p* = 0.0010; genotype: *F* = 0.7167, *p* = 0.4359. For small diameters, WT: 59.95 ± 0.4234%, *Itpr2^–/–^*: 76.34 ± 4.492%; for large diameters, WT: 7.68 ± 0.9467%, *Itpr2^–/–^*: 0.13 ± 0.07336%). Similarly, the proportion of small-diameter myelinated axons in the *Itpr2* mutant optic nerve also increased significantly, while the large-diameter myelinated axons decreased (Figure 3D; Two-way ANOVA *P*-values summary: interaction: *F* = 11.61, *p* < 0.0001; axon diameter: *F* = 111.1, *p* < 0.0001; genotype: *F* = 0.5946, *p* = 0.4837. For small diameters, WT: 28.71 ± 0.3378%, *Itpr2^–/–^*: 43.35 ± 0.9538%; for large diameters, WT: 14.48 ± 1.055%, *Itpr2^–/–^*: 6.33 ± 1.253%). Thus, *Itpr2* mutation significantly reduced the diameters of myelinated axons. As a whole group, g-ratios were unaltered in the corpus callosum and optic nerves between the two groups of animals, but the g-ratios in different diameter range displayed different trends. As the scatter plots of g-ratio against axon caliber showed the small-diameter myelinated axons exhibited smaller g-ratios indicative of thicker myelin sheath in the mutant CC and optic nerves, while the large-diameter myelinated axons showed the opposite result (Figures 3E,F). Together, these data manifest that *Itpr2* deficiency altered the size population of myelinated axons in the CNS, increasing the percentage of smaller axons for myelination.

### Reduced Conduction Velocity in the *Itpr2* Deficient Central Nervous System

**FIGURE 4:**
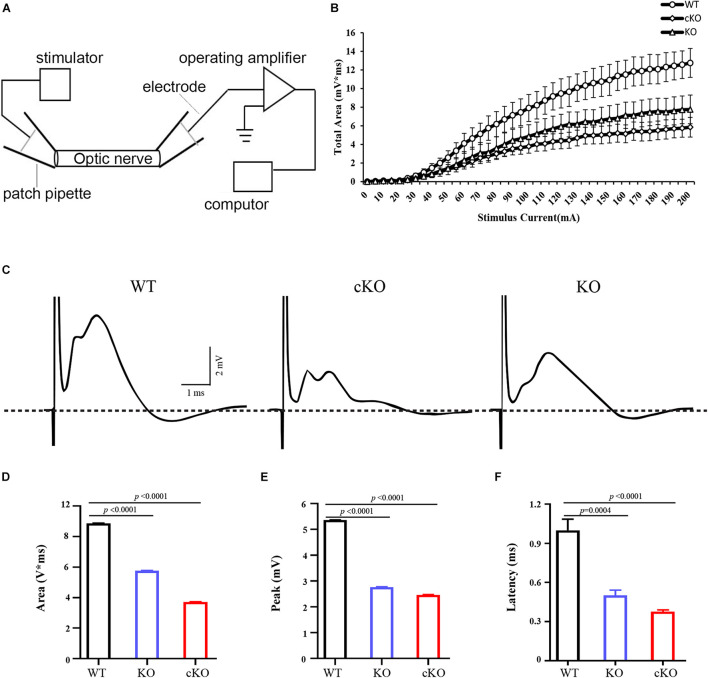
Electrophysiological measurements of CAP from optic nerves. **(A)** The diagram of electrophysiological measurements of CAP from optic nerve. **(B)** Normalized total CAP area of optic nerves showing a S-type growth in response to increasing stimulus current (*n* = 5, 7, 4). **(C)** Representative 70% maximum CAP of optic nerves from wild-type (WT), conditional knockout (*cKO*) and conventional mutants (*KO*) mice. The large stimulus artifact has been truncated and is followed by a polyphasic waveform peaks. And compared to the control groups, the first peak became smaller and the third peak became larger in the *Itpr2 cKO* and *KO* groups. **(D)** Analysis of the values for the CAP area (WT: 8.878 ± 0.01151 mV*ms, *KO*: 5.759 ± 0.03151 mV*ms, *cKO*: 3.713 ± 0.01563 mV*ms, one-way ANOVA, *F* = 14817, *p* < 0.0001, *n* = 6, 6, 6.) in the optic nerves. **(E)** Analysis of the values for CAP peak (WT: 5.364 ± 0.01007 mV, *KO*: 2.763 ± 0.01365 mV, *cKO*: 2.464 ± 0.01118 mV, one-way ANOVA, *F* = 18485, *p* < 0.0001, *n* = 6, 6, 6) in the optic nerves. **(F)** Analysis of the values for CAP latency (WT: 1.000 ± 0.08515 ms, *KO*: 0.5000 ± 0.04082 ms, *cKO*: 0.3750 ± 0.01443 ms, one-way ANOVA, *F* = 29.99, *p* < 0.0001, *n* = 5, 4, 4.) in the optic nerves. Error bars indicate Means ± SEM.

Previous studies showed CAP recorded from rodent optic nerve is polyphasic in profile, with total area under the CAP as an index of nerve function. It is suggested that the CAP is related to the composition of the axons (Evans et al., 2010). To further confirm our results, we recorded the CAP of optic nerves from wild-type, *Itpr2^–/–^* and *Itpr2cKO* mice (Figure 4A). With the increase of stimulation intensity, the total CAP area increased continuously and finally reached a stable plateau for control, *Itpr2cKO* and *KO* groups (Figure 4B). However, the total CAP area was significantly decreased in *Itpr2cKO* and *KO* mice compared to wild-type mice (Figure 4D; One-way ANOVA, *F* = 18485, *p* < 0.0001). The typical evoked CAP response was polyphasic in profile (Figure 4C), which is in line with the previous findings (Govind and Lang, 1976; Evans et al., 2010; Horowitz et al., 2015). Prior studies showed that CAP could exhibit multiple peaks, with the largest diameter axons contributing to the 1st CAP peak and the smaller axons contributing to the 2nd and 3rd CAP peaks (Evans et al., 2010). Thus, the shift in the relative proportion of small vs. large axons would suggest that the peaks of the axons shift in size. Compared to the control groups, the first peak became smaller in both *Itpr2cKO* and *KO* groups (Figure 4E; One-way ANOVA, *F* = 18485, *p* < 0.0001), in agreement with our electron microscopy results (Figure 3). We also found that the values for the latency for the 1st peak increased in the *Itpr2cKO* and *KO* mice compared to wild-type mice (Figure 4F; One-way ANOVA, *F* = 29.99, *p* < 0.0001). Collectively, these data indicated that a higher proportion of smaller myelinated fibers in *Itpr2* mutant mice adversely affected their CAP in the CNS.

### Increased Percentage of Type I/II Oligodendrocytes Was Increased in *Itpr2* Mutants

**FIGURE 5:**
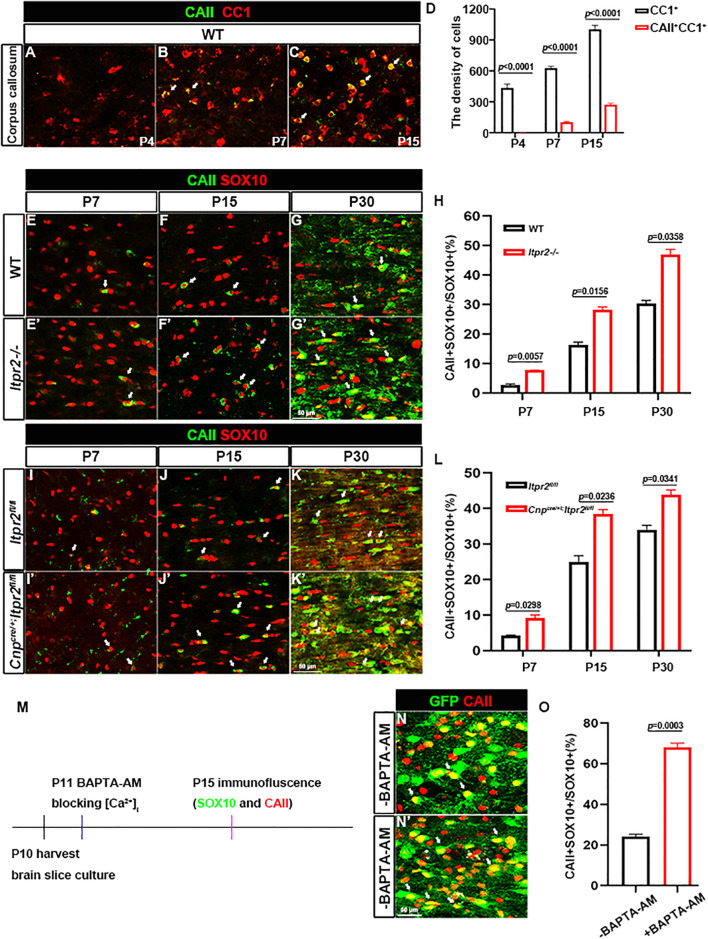
Disruption of ITPR2 increases the proportion of type I/II oligodendrocytes. **(A–C)** Double immunostaining of CAII and CC1 in early postnatal brain tissues. **(D)** Quantification of the density of CC1 positive cells and CAII/CC1 double positive cells in the corpus callosum of wild-type mice. **(E–G′)** Immunofluorescent images of CAII and SOX10 staining in corpus callosum tissues from various stages of WT and *Itpr2-/*mice. Double positive cells are represented by white arrows. **(H)** Quantitative analysis of the ratio of CAII^+^ cells in SOX10^+^ oligodendrocytes at indicated stages. **(I–K′)** CAII and SOX10 double-immunostaining in corpus callosum tissues from various stages of control and *Cnp^*cre/*+^*; *Itpr2^*fl/fl*^* mice. Double positive cells are represented by white arrows. Scale bar represents 50 μm. **(L)** Quantitative analysis of the percentage of CAII^+^ cells in SOX10^+^ oligodendrocytes at indicated stages. **(M)** Schematic of workflow of organotypic slice cultures. **(N,N′)** Immunostaining of CAII in the corpus callosum of cultured *Sox10-GFP* slices. Compared with the control groups, the ratio of CAII^+^SOX10^+^ oligodendrocytes remarkably increased in the corpus callosum of cultured slices treated with 20 μM BAPTA-AM. Scale bar represents 50 μm. **(O)** Quantification of the percentage of CAII/SOX10-GFP double positive cells in SOX10-GFP^+^ population in brain slice culture from **(N,N′)**. Error bar indicates Means ± SEM. *n* ≥ 3.

We next explored the possible mechanism underlying the increased percentage of small caliber axons that are myelinated in the mutants. Early studies identified four types of oligodendrocytes, among which CAII^+^ type I/II OLs tend to myelinate small caliber axons (Butt et al., 1998; Butt and Berry, 2000). Thus, we next investigated the possibility that the delayed OL differentiation in the mutants may cause an increased proportion of type I/II OLs, leading to a higher percentage of smaller myelinated axons. Immunostaining of the wild-type brain tissues revealed that at P4, CAII^+^/CC1^+^ double positive cells were rarely seen in the corpus callosum (Figure 5A). At P7, a small number of CAII^+^ positive cells began to emerge in the corpus callosum (Figure 5B). By P15, the density of CAII^+^/CC1^+^ type I/II cells was significantly increased in the corpus callosum (Figures 5C,D), suggesting that the type I/II group were later-born OLs. Consistently, the percentage of CAII^+^SOX10^+^ OLs in SOX10^+^ population (white arrows) in *Itpr2* mutant mice was significantly elevated in the white matter at all postnatal stages examined (Figures 5E–H). A similar increase in the ratio of CAII^+^SOX10^+^ OLs was also found in the *Itpr2* conditional mutant (*cKO*) brain tissues (Figures 5I–L). Together, these data strongly suggest that the delayed OL differentiation in *Itpr2* deficiency results in an increased ratio of type I/II oligodendrocytes.

Given that ITPR2 is the main receptor for intracellular release of calcium, it is plausible that Ca^2+^ signaling may be involved in the differentiation of OL subtypes. To address this possibility, membrane permeable Ca^2+^ chelator BAPTA-AM was applied to brain slice culture of *Sox10-GFP* mice to block intracellular calcium release. After 5 days of treatment, brain slices were subjected to CAII immunostaining (Figure 5M). Consistent with the *in vivo* findings, the density of CAII^+^ type I/II OLs was significantly increased after treatment with 20 μm BAPTA-AM (Figures 5N,N′,O). Thus, disruption of ITPR2 gene and blocking intracellular calcium release had the same effect of significantly increasing the proportion of type I/II oligodendrocytes both *in vivo* and *in vitro*.

**FIGURE 6:**
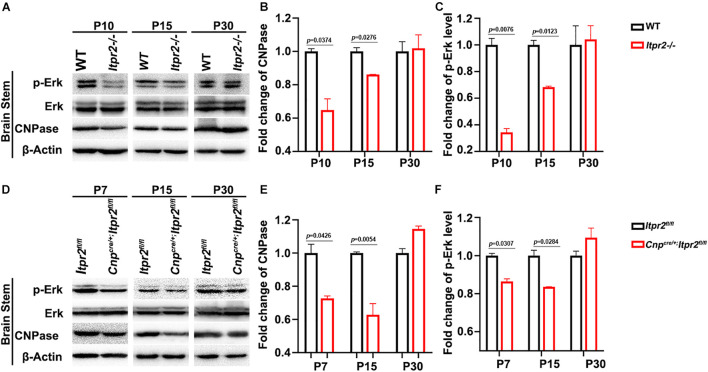
*Itpr2* deletion inhibits the Ca^2+^-dependent ERK activation. **(A)** The expression and phosphorylation of ERK, CNPase and ERK in brainstem tissues from wild-type and *Itpr2^–/–^* mice at P10, P15 and P30 in grouping image. β-actin was used as the internal control. **(B)** Quantification of CNPase protein level in the brainstem of *Itpr2^–/–^* mice which were normalized to the wild-type group. **(C)** Quantification of ERK1/2 phosphorylation level in the brainstem of *Itpr2^–/–^* mice which were normalized to the wild-type group. **(D)** The expression and phosphorylation of ERK, CNPase, and ERK in brainstem tissues from control and *Cnp^*cre/*+^*; *Itpr2^*fl/fl*^* mice at P7, P15, and P30 in grouping image. β-actin was used as the internal control. **(E)** Quantification of CNPase protein level in the brainstem of *Cnp^*cre/*+^*; *Itpr2^*fl/fl*^* mice which were normalized to the control group. **(F)** Quantification of ERK phosphorylation level in the brainstem of *Cnp^*cre/*+^*; *Itpr2^*fl/fl*^* mice which were normalized to the control group. Error bar indicates Means ± SEM. *n* = 3.

The ERK1/2 pathway has been shown to be critical for OL differentiation both *in vitro* and *in vivo* (Guardiola-Diaz et al., 2012; Chen et al., 2015; Rodgers et al., 2015; Mei et al., 2021). Previous studies have shown that the release of calcium from intracellular stores can stimulate the ERK activity which can directly influence OL differentiation (Kim et al., 2020). Consequently, we conjectured whether preventing the release of intracellular calcium influx by deletion of ITPR2 can down-regulate the ERK phosphorylation level. To test this possibility, we examined the expression of CNPase, ERK and p-ERK via Western blotting (Figures 6A,D), and found a decrease of CNPase expression and p-ERK level in the brainstem of *Itpr2cKO* and *KO* mice at P7 and P15, while the total level of ERK protein was not significantly altered (Figures 6B,C,E,F). Thus, the expression of CNPase and ERK phosphorylation level was indeed down-regulated in the conventional and conditional mutant brainstem at early developmental stages. Taken together, our studies suggest that ITPR2 deletion may affect the differentiation of oligodendrocytes and the ratio of type I/II oligodendrocytes by abating Ca^2+^-dependent ERK activation.

## Discussion

Oligodendrocytes arise from specific regions of neural epithelium and then migrate to the entire CNS before they differentiate and form myelin sheaths wrapping around neuronal axons. These progressive processes are accurately controlled by a large number of intracellular factors and extracellular signals. Calcium signaling emerges as an important regulator for oligodendrocyte development and axonal myelination (Soliven, 2001). There are three IP_3_R subtypes (ITPR1-3) in mammals (Furuichi et al., 1994). We found that only ITPR2 is highly expressed in differentiating OLs, consistent with the recent studies with RNA-seq analyses showing that *Itpr2* is expressed in cells of oligodendrocyte lineage (Zeisel et al., 2015; Marques et al., 2016). Expression of the other two isoforms is not detected in the white matter in both control and *Itpr2^–/–^* spinal tissues (Supplementary Figure 3), indicating a lack of compensatory up-regulation of *Itpr1*/*3* in the absence of *Itpr2.* Based on these findings, we believe that ITPR2 is the predominant channel responsible for intracellular release of calcium in OLs.

Our expression analyses established that *Itpr2* is highly up-regulated in differentiating OLs at early postnatal stages (Figure 1 and Supplementary Figure 1) when oligodendrocytes undergo active differentiation and myelination (Franco-Pons et al., 2006; Korrell et al., 2019). In fact, its expression is not detected in immature OPCs in embryonic tissues and is downregulated after axonal myelination in adult tissues (Figure 1 and Supplementary Figure 1). The strong expression of *Itpr2* in differentiating OLs suggests its important role in regulating OL differentiation and myelin formation. In support of this idea, *Itpr2* deficiency induced a transient developmental delay of OL differentiation and myelin gene expression (Figure 2). The delayed OL differentiation in the mutants is apparently associated with the abnormal myelin sheath development and axonal function, as suggested by the marked increase in the percentage of small diameter myelinated axons in the corpus callosum and optic nerve, and the abnormal CAP in *Itpr2* knockout (Figures 3, 4). Since our expression analyses clearly demonstrate that *Itpr2* is not expressed in neurons or astrocytes at the early postnatal stages when oligodendrocyte differentiating, we would argue that the phenotypic alterations observed in both conventional and conditional mutants are cell-autonomous and attributed to defects in oligodendrocyte development. Consistent with this idea, deleting *Itpr2* in conditional mutants does not appear to affect the expression of astrocyte markers in the corpus callosum or neuronal markers in the cortex (Supplementary Figure 4).

It was previously demonstrated that type I/II oligodendrocytes predominantly myelinate small diameter axons, and these myelin sheaths display fewer lamellae and shorter internodal lengths (Butt and Berry, 2000). In keeping with this early finding, we detected an increased population of type I/II OLs in *Itpr2* mutant brain tissues (Figure 5). Meanwhile, the ratio of small diameter myelinated axons to larger ones is increased in the mutant tissues, which negatively impacts the saltatory conduction of electrical signals. It has been previously demonstrated that the generation of the precise number of OLs is necessary to myelinate entirely a given population of axons (Barres et al., 1992; Burne et al., 1996; Barres and Raff, 1999), and that small fiber diameter (0.2–0.4 μm) is sufficient to initiate wrapping by oligodendrocytes (Lee et al., 2012). It is conceivable that an excess number of type I/II OLs shifts myelination to smaller diameter axons, and the ratio of small/large diameter myelinated axons was altered together with the conduction velocity of electrical signals. A similar observation was made in *Gab1^*f*/f^; Olig1*^*cre*/+^ mice, in which ablation *Gab1* in the OLs resulted in delayed OL differentiation and an increased proportion of small-diameter axons being myelinated (Zhou et al., 2020). At this stage, it is not known why type I/II OLs are associated with small caliber axons. One possibility is that these late-born OLs fail to provide certain trophic or nourishing factors for further growth of myelinated axons. Alternatively, ITPR2 deficiency may affect the formation of Ca^2+^ wave which in turn affects the neuronal activity that has been suggested to regulate the sizes of axon (Sinclair et al., 2017). In addition, earlier reports have showed that ERK signaling pathway is necessary for oligodendrocyte differentiation (Sun et al., 2013; Gaesser and Fyffe-Maricich, 2016). Interestingly, the release of calcium from intracellular stores can stimulate the ERK activity (Kim et al., 2020). Our data have shown that *Itpr2* expression is correlated with the elevation of ERK phosphorylation (Figure 6), raising the possibility that ITPR2 might regulate oligodendrocyte differentiation via the intracellular calcium-dependent activation of the ERK pathway.

In summary, our studies provide the important molecular and genetic evidence that *Itpr2* is dramatically up-regulated in differentiating OLs and regulates OL differentiation and myelin development. To our knowledge, this is the first report that ITPR2-mediated calcium signaling can directly affect the differentiation of OL subtypes possibly through an ERK-dependent mechanism, and therefore influence the development of myelinated axons.

## Data Availability Statement

The raw data supporting the conclusions of this article will be made available by the authors, without undue reservation.

## Ethics Statement

The animal study was reviewed and approved by the Animal Ethics Committee of Hangzhou Normal University, China.

## Author Contributions

MQ and XZ conceived the main ideas and supervised the project. RM and LH performed most of the experimental operations. MW and CJ carried out the main parts of the numerical calculations. RM, AY, and HT carried out the rest of them. KZ, JY, WS, and XC conceived the experiments and supervised this research. All authors discussed and interpreted the results, and reviewed the manuscript.

## Conflict of Interest

The authors declare that the research was conducted in the absence of any commercial or financial relationships that could be construed as a potential conflict of interest.

## Publisher’s Note

All claims expressed in this article are solely those of the authors and do not necessarily represent those of their affiliated organizations, or those of the publisher, the editors and the reviewers. Any product that may be evaluated in this article, or claim that may be made by its manufacturer, is not guaranteed or endorsed by the publisher.

## Supplementary Material

Supplementary Figure 1 Expression pattern of ITPR2 in the spinal cord. **(A–F)** *Itpr2* ISH in mouse spinal cord from E18.5 to P30. Scale bar represents 25 μm. Black arrows highlight *Itpr2*^+^ cells. **(G–J)** ITPR2 double immunofluorescence with anti-OLIG2, anti-CC1 or anti- NeuN in P4, and P7 spinal cord sections. ITPR2 positive cells are mostly co-labeled with OLIG2 and CC1 (white arrows), but not with anti-NeuN. Scale bar represents 50 μm.

Supplementary Figure 2 Expression of *Itpr2* in *Nkx2.*2-*cKO* **(A,B)** and *Myrf*-*cKO.* **(C,D)** Mutant spinal cords is dramatically reduced at P3 stages. Scale bar represents 100 μm.

Supplementary Figure 3 Disruption of *Itpr2* has no effect on *Itpr1* and *Itpr3* expression in the white matter. Spinal cord tissues from P3 **(A–D)** and P7 **(E–H)** wild-type and *Itpr2^–/–^* mice were examined for expression of *Itpr1* and *Itpr3* by ISH. Scale bar represents 100 μm.

Supplementary Figure 4 ITPR2 deletion produces no difference to the astrocytes in the corpus callosum and neurons in the cortex. Brain tissues from P15 wild-type and *Itpr2* knockout mice were immunostaining with astrocyte marker GFAP **(A,B)** and neuron marker NeuN **(C,D)**. Scale bar represents 50 μm.

Supplementary Figure 5 Analyses of the number of myelinated axons in the corpus callosum **(A)** and optic nerves **(B)** from wild-type and *Itpr2-/–* mice. Error bar indicates Means ± SEM. *n* ≥ 3.
