# Supplementary figures and images for: Evidence That ITPR2-Mediated Intracellular Calcium Release in Oligodendrocytes Regulates the Development of Carbonic Anhydrase II + Type I/II Oligodendrocytes and the Sizes of Myelin Fibers
Source: Front Cell Neurosci. 2021 Sep 22;15:751439. doi: 10.3389/fncel.2021.751439 (PMC8492996; doi:10.3389/fncel.2021.751439)

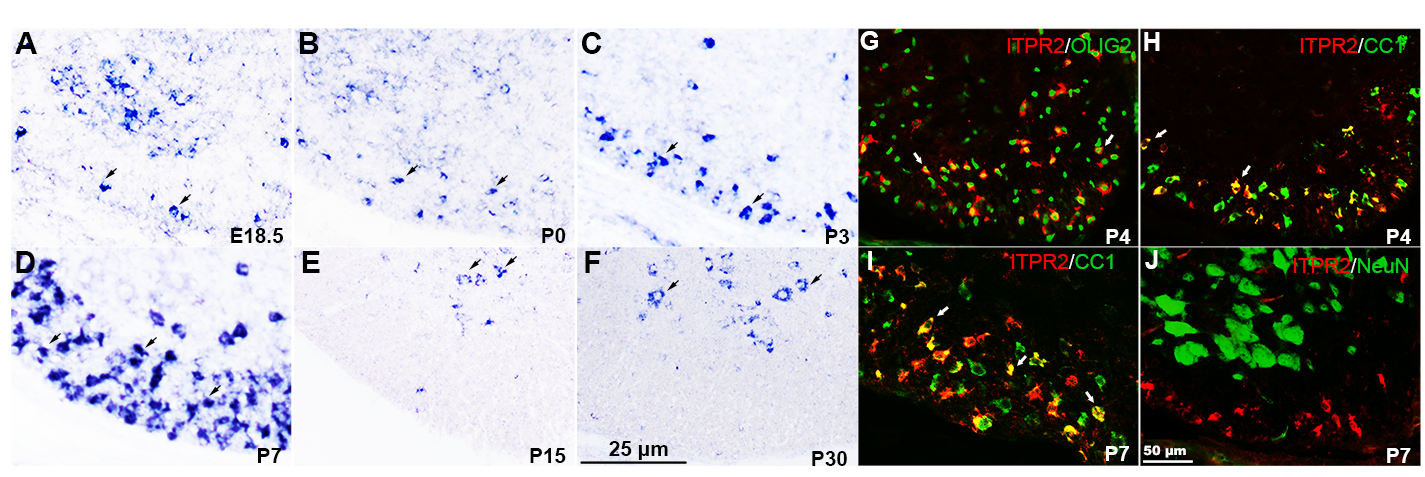

Supplement: Supplementary Figure 1 — Expression pattern of ITPR2 in the spinal cord. (A–F) Itpr2 ISH in mouse spinal cord from E18.5 to P30. Scale bar represents 25 μm. Black arrows highlight Itpr2+ cells. (G–J) ITPR2 double immunofluorescence with anti-OLIG2, anti-CC1 or anti- NeuN in P4, and P7 spinal cord sections. ITPR2 positive cells are mostly co-labeled with OLIG2 and CC1 (white arrows), but not with anti-NeuN. Scale bar represents 50 μm. [file Image_1.TIF]

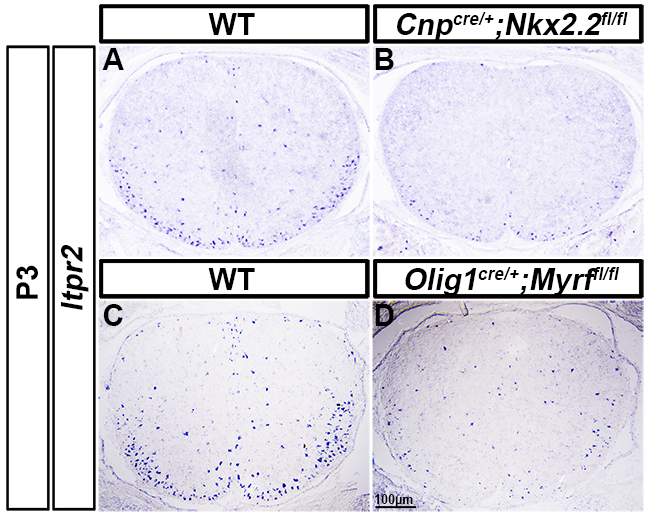

Supplement: Supplementary Figure 2 — Expression of Itpr2 in Nkx2.2-cKO (A,B) and Myrf-cKO. (C,D) Mutant spinal cords is dramatically reduced at P3 stages. Scale bar represents 100 μm. [file Image_2.TIF]

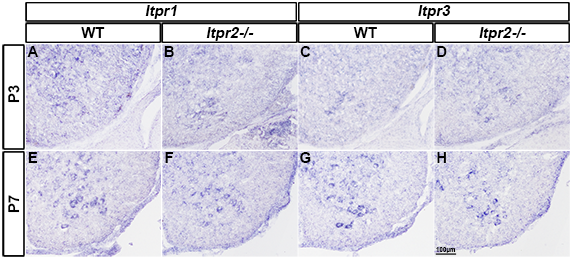

Supplement: Supplementary Figure 3 — Disruption of Itpr2 has no effect on Itpr1 and Itpr3 expression in the white matter. Spinal cord tissues from P3 (A–D) and P7 (E–H) wild-type and Itpr2–/– mice were examined for expression of Itpr1 and Itpr3 by ISH. Scale bar represents 100 μm. [file Image_3.TIF]

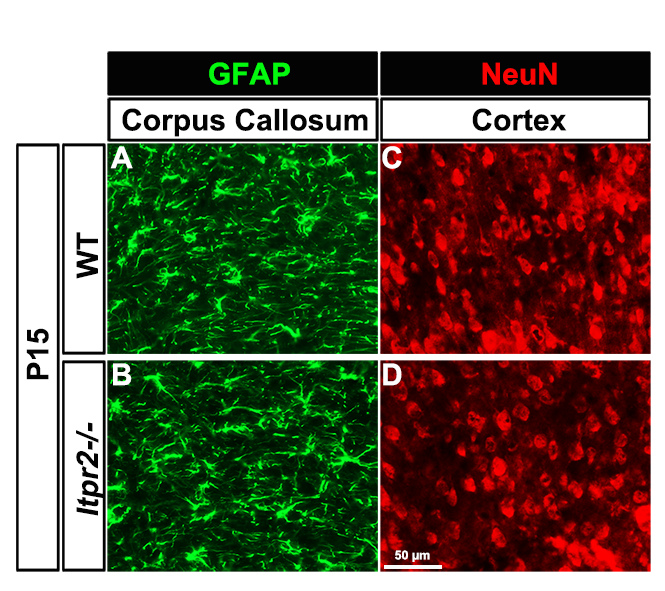

Supplement: Supplementary Figure 4 — ITPR2 deletion produces no difference to the astrocytes in the corpus callosum and neurons in the cortex. Brain tissues from P15 wild-type and Itpr2 knockout mice were immunostaining with astrocyte marker GFAP (A,B) and neuron marker NeuN (C,D). Scale bar represents 50 μm. [file Image_4.TIF]

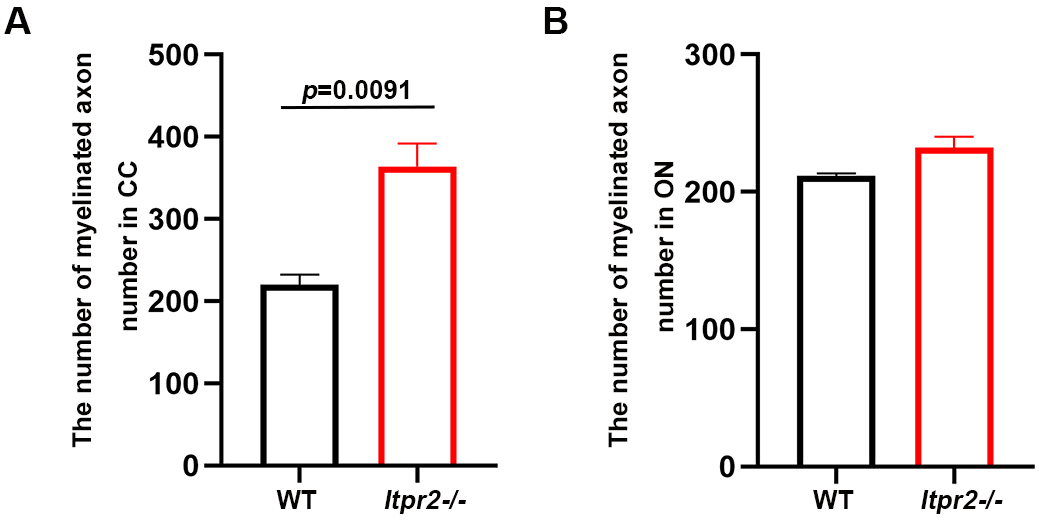

Supplement: Supplementary Figure 5 — Analyses of the number of myelinated axons in the corpus callosum (A) and optic nerves (B) from wild-type and Itpr2-/– mice. Error bar indicates Means ± SEM. n ≥ 3. [file Image_5.TIF]
